# Neurobehavioral assessment of a syndromic Dandy-Walker malformation using 4D ultrasound and KANET: a case report

**DOI:** 10.1515/crpm-2025-0008

**Published:** 2025-09-23

**Authors:** Wiku Andonotopo, Muhammad Adrianes Bachnas, Julian Dewantiningrum, Mochammad Besari Adi Pramono, Milan Stanojević, Asim Kurjak

**Affiliations:** Department of Obstetrics and Gynecology, Maternal-Fetal Medicine Division, Women Health Center, Eka Hospital, Tangerang, Banten, Indonesia; Department of Obstetrics and Gynecology, Maternal-Fetal Medicine Division, Faculty of Medicine Sebelas Maret University, Dr. Moewardi General Hospital, Solo, Indonesia; Department of Obstetrics and Gynecology, Maternal-Fetal Medicine Division, Medical Faculty of Diponegoro University, Dr. Kariadi Hospital, Semarang, Indonesia; Department of Neonatology and Rare Diseases, Medical University of Warsaw, Warsaw, Poland; Department of Obstetrics and Gynecology, Medical School University of Zagreb, Zagreb, Croatia

**Keywords:** Dandy-Walker malformation, fetal neurobehavior, KANET, 4D ultrasound

## Abstract

**Objectives:**

To evaluate fetal neurobehavior using the Kurjak Antenatal Neurodevelopmental Test (KANET) via 4D ultrasound in a fetus diagnosed with syndromic Dandy-Walker Malformation (DWM), and to correlate these findings with postnatal outcomes.

**Case presentation:**

A 35-year-old woman was referred at 25 weeks’ gestation for evaluation of multiple fetal anomalies. Ultrasound revealed hallmark DWM features – cerebellar vermis hypoplasia, enlarged posterior fossa, hydrocephalus – as well as extracranial anomalies including craniofacial dysmorphism, bilateral clubhands and clubfeet, and suspected hypoplastic left heart syndrome. A KANET assessment at 25+3 weeks yielded a severely abnormal score of 3. At 26 weeks, a male infant was delivered and survived 2 h postnatally. All anomalies were confirmed postnatally.

**Conclusions:**

KANET, even when performed slightly earlier than standard timing, provided critical insights into the fetus’s neurobehavioral function and correlated strongly with the fatal outcome. This case supports the value of KANET as a non-invasive tool for assessing neurological integrity in complex fetal conditions where third-trimester evaluation may not be possible.

## Introduction

Dandy-Walker Malformation (DWM) is a congenital anomaly of the central nervous system (CNS), characterized by hypoplasia or agenesis of the cerebellar vermis, cystic dilation of the fourth ventricle, and enlargement of the posterior fossa. These hallmark features are often accompanied by other supratentorial abnormalities, including hydrocephalus, corpus callosum dysgenesis, and brainstem malformations [1], 2]. In syndromic or chromosomal forms, DWM is frequently associated with extracranial anomalies such as craniofacial dysmorphism, congenital heart defects, and limb malformations.

While prenatal imaging modalities like ultrasound and fetal MRI are effective in detecting structural CNS abnormalities, they provide limited insight into functional neurological status. Assessing fetal neurodevelopment *in utero* remains a clinical challenge, particularly in complex or high-risk pregnancies.

The Kurjak Antenatal Neurodevelopmental Test (KANET) is a standardized screening tool designed to evaluate fetal neurobehavior using four-dimensional (4D) ultrasound [3], 4]. It assesses eight behavioral parameters – including facial expressions, isolated and general limb movements, and finger activity – to generate a composite score reflecting CNS function. KANET is non-invasive and has shown utility in identifying fetuses at risk for neurodevelopmental impairment, particularly in pregnancies complicated by CNS anomalies, growth restriction, or chromosomal abnormalities [4], [5], [6], [7], [8].

Although KANET is typically performed between 28 and 38 weeks of gestation, recent literature supports its use in earlier gestational windows when clinically indicated. Studies have shown that even when applied before 28 weeks, KANET can yield meaningful insights in high-risk cases, including those with early-onset malformations or impending preterm delivery [3], 5], 9].

The objective of this case report is to evaluate the clinical application of KANET as a functional assessment tool in a fetus diagnosed with syndromic DWM and multiple congenital anomalies. By correlating abnormal prenatal neurobehavioral findings with the confirmed postnatal outcome, this report illustrates the prognostic value of KANET and its potential role in early, multidisciplinary prenatal evaluation. In doing so, it contributes to the growing literature supporting KANET as a complementary tool alongside structural imaging for informed perinatal decision-making.

## Case presentation

A 35-year-old gravida 3, para 1 woman was referred at 25 weeks of gestation to the maternal-fetal medicine unit after routine ultrasound identified multiple fetal anomalies. The pregnancy was naturally conceived. Her medical, surgical, and obstetric histories were unremarkable, with no consanguinity, no prior congenital anomalies, and no known exposure to teratogens. First-trimester screening at 12 weeks revealed increased nuchal translucency (5.0 mm), but non-invasive prenatal testing (NIPT) at 13 weeks indicated a normal male karyotype (46,XY). Maternal serologies, including TORCH screening, were negative.

**Figure 1:**
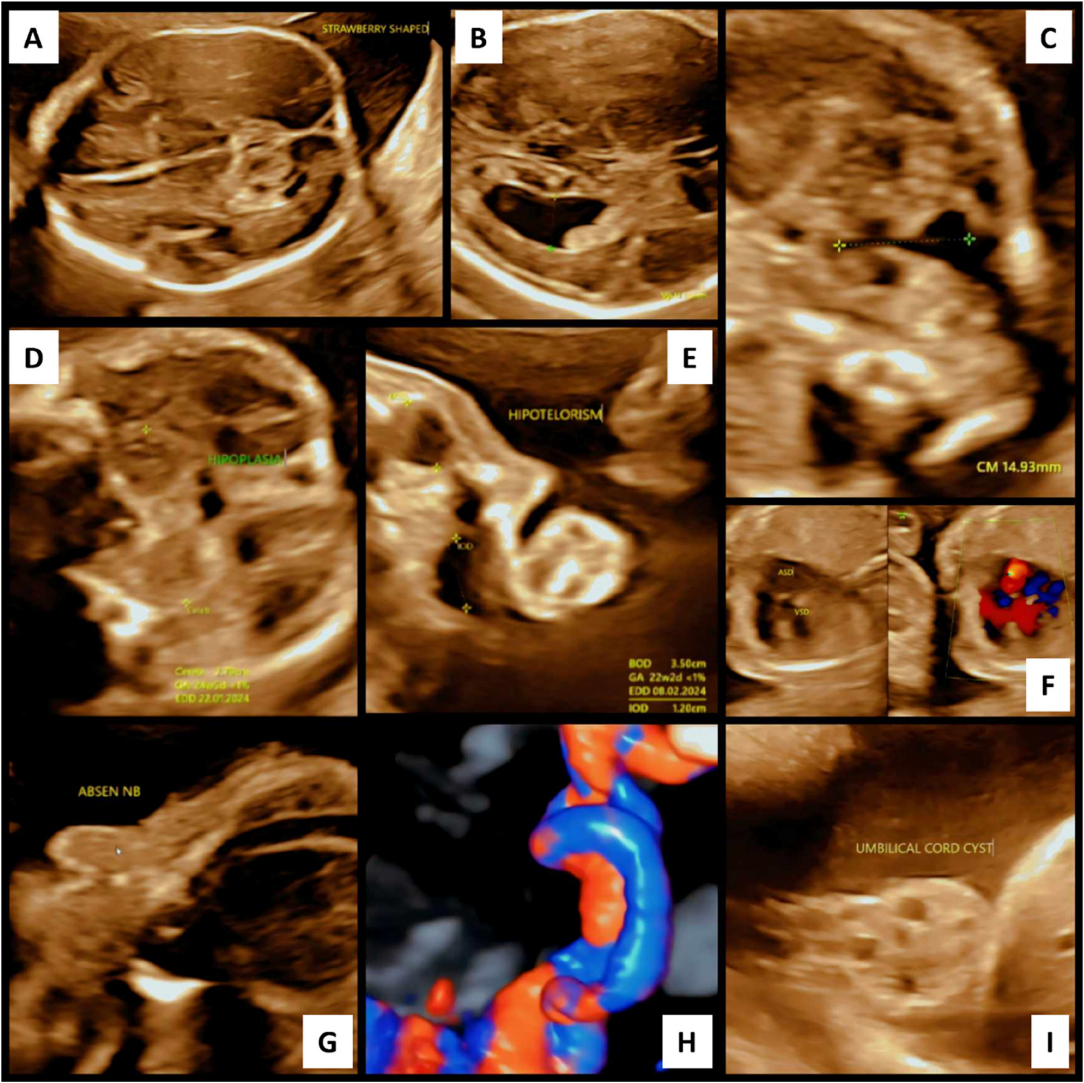
2D ultrasound features of syndromic Dandy-Walker malformation with multisystem anomalies. (A) The fetal head shows a mild strawberry shape with posterior flattening and frontal narrowing. (B) Lateral ventriculomegaly indicates hydrocephalus. (C) An enlarged cisterna magna and dilated posterior fossa are consistent with Dandy-Walker malformation. (D) Cerebellar vermis is hypoplastic. (E) Reduced biocular diameter reflects hypotelorism. (F) Echocardiography suggests hypoplastic left heart syndrome with a small left ventricle and retrograde aortic flow. (G) Flattened nasal bridge and absent/hypoplastic nasal bone indicate midface hypoplasia. (H) Single umbilical artery is seen on cord cross-section. (I) A well-defined umbilical cord cyst is visible.

A detailed anomaly scan at 18 weeks revealed central nervous system, craniofacial, limb, and cardiac anomalies. The fetal head was mildly flattened posteriorly, with a strawberry-like configuration. Neurosonography demonstrated an enlarged posterior fossa, cystic dilation of the fourth ventricle communicating with an expanded cisterna magna, and cerebellar vermis hypoplasia – findings consistent with Dandy-Walker Malformation (DWM). Ventriculomegaly involving the lateral and third ventricles suggested progressive hydrocephalus. Head circumference measured above the 95th percentile. Representative 2D ultrasound images are shown in Figure 1.

**Figure 2:**
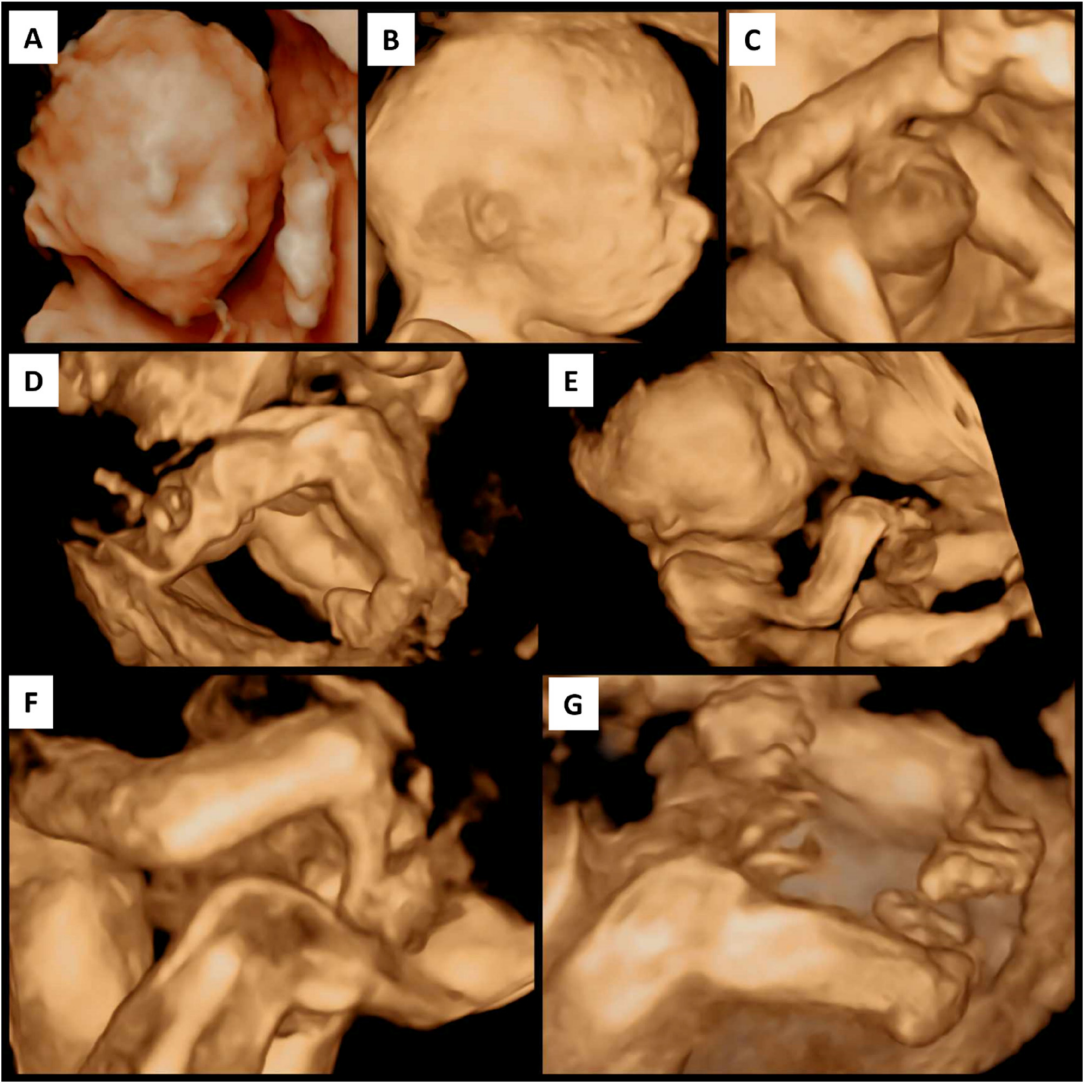
Shows the standard 3D ultrasound appearance in a fetus with syndromic Dandy-Walker malformation. The face appears rounded with midface hypoplasia, indicating facial deformity (A), while the right ear is visibly low-set and hypoplastic, consistent with microtia (B). A distinct umbilical cord cyst is noted along the cord (C). The lower limbs show bilateral clubfoot with persistent medial rotation and plantar flexion (D), and the overall fetal body displays abnormal posture with bilateral clubhand deformities, including shortened forearms and inward wrist deviation (E). The upper limbs confirm radial clubhand on both sides (F), and the feet again demonstrate fixed bilateral clubfoot positioning (G), all reflecting severe multisystem anomalies associated with neurodevelopmental impairment.

Craniofacial findings included hypoplastic, low-set auricles (microtia), a flattened nasal bridge, and hypoplastic nasal bones. No cleft lip or palate was present. Limb anomalies included bilateral radial clubhands, shortened forearms, and inward deviation of the wrists, consistent with limb reduction defects. Bilateral talipes equinovarus (clubfoot) with persistent medial rotation and plantar flexion was also noted. Figure 2 presents complementary 3D ultrasound images illustrating these dysmorphic features and abnormal fetal posture.

Fetal echocardiography raised suspicion for hypoplastic left heart syndrome (HLHS), with a diminutive left ventricle, poorly visualized mitral and aortic valves, and suspected retrograde aortic arch flow – suggesting a functionally univentricular heart incompatible with long-term survival without major intervention.

**Figure 3:**
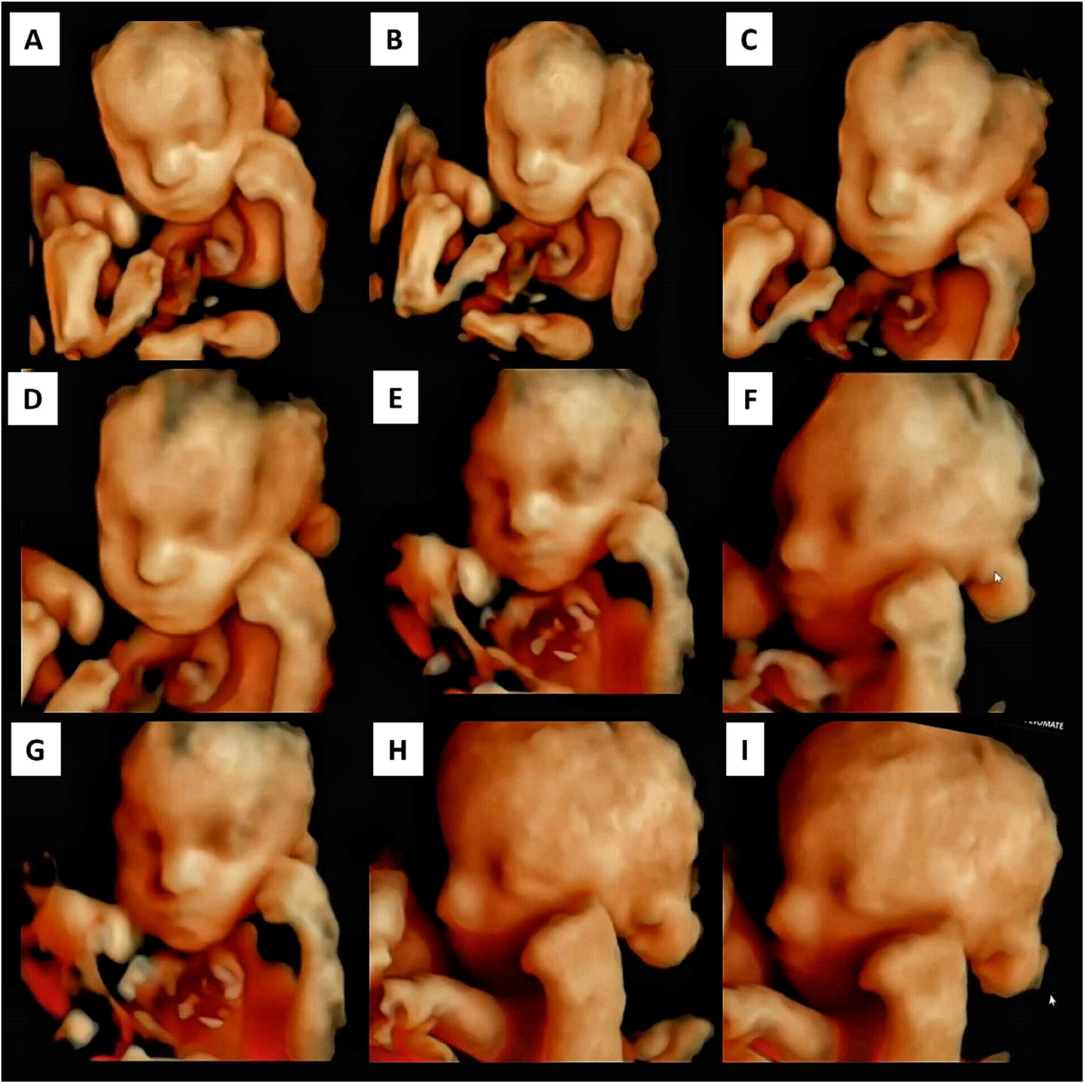
4D AI-enhanced ultrasound with KANET in syndromic Dandy-Walker malformation. This AI-enhanced 4D ultrasound shows severely abnormal fetal neurobehavior in a case of syndromic Dandy-Walker malformation. The fetus scored three on the KANET scale, indicating major neurological dysfunction. Movements were minimal, uncoordinated, and lacked variability. There were no facial expressions, eye blinking, or goal-directed activity. The overall pattern reflects severe CNS impairment and correlates with the poor postnatal outcome. AI enhancement was used to reduce background noise and improve contrast for better visualization of subtle neurobehavioral movements during 4D ultrasound.

At 25 weeks and 3 days, a fetal neurobehavioral assessment was performed using a 4D ultrasound system (Voluson E-Series, GE Healthcare) following the Kurjak Antenatal Neurodevelopmental Test (KANET) protocol. A 30-min continuous observation revealed severely abnormal neurobehavior: absent facial expressions (e.g., mouthing, blinking), reduced and monotonous limb movements, fixed clenched fists, poor variability, and no hand-to-face or cranio-directed activity. General movements lacked fluency and complexity, and the fetus remained in a rigid posture throughout. Finger activity was cramped and inflexible. The total KANET score was 3, indicating a profoundly abnormal neurodevelopmental profile and severe CNS dysfunction. Full scoring details are provided in Table 1. AI-enhanced 4D ultrasound images depicting these findings are presented in Figure 3.

**Table 1:** KANET scoring analysis for the fetus with syndromic Dandy-Walker malformation.

KANET parameter	Score	Observation
Isolated head anteflexion	0	Head movement not assessable due to hydrocephalus and cerebellar vermis hypoplasia; no spontaneous anteflexion observed
Cranial sutures and head circumference	0	Abnormal: macrocephaly due to hydrocephalus; head circumference>95th percentile; cranial configuration altered
Isolated eye blinking	0	No eyelid or blinking activity observed throughout the 30-min scan
Facial alterations (grimacing, smiling, mouthing)	0	Absent facial movements; no observable expressions such as yawning, mouthing, or grimacing
Isolated hand movements	1	Severely reduced hand movements; both hands remained clenched anterior to the chest with poor variability and minimal activity
Isolated leg movements	1	Reduced amplitude and frequency; movements lacked complexity and flexibility; no alternating or variable leg motion was recorded
Hand-to-face movements	0	Absent; no attempts of hand-to-face or cranio-directed activity during the observation period
Finger movements	1	Cramped, monotonous, and uncoordinated finger activity; hands often remained clenched without extension or differentiation of finger movements
Gestalt perception of general movements	0	Overall movement pattern was monotonous, abrupt, and lacked fluency; general posture remained fixed with no spontaneous repositioning
→ Total KANET score	3	Abnormal (0–5): Indicates severe neurodevelopmental impairment and high likelihood of central nervous system dysfunction

**Figure 4:**
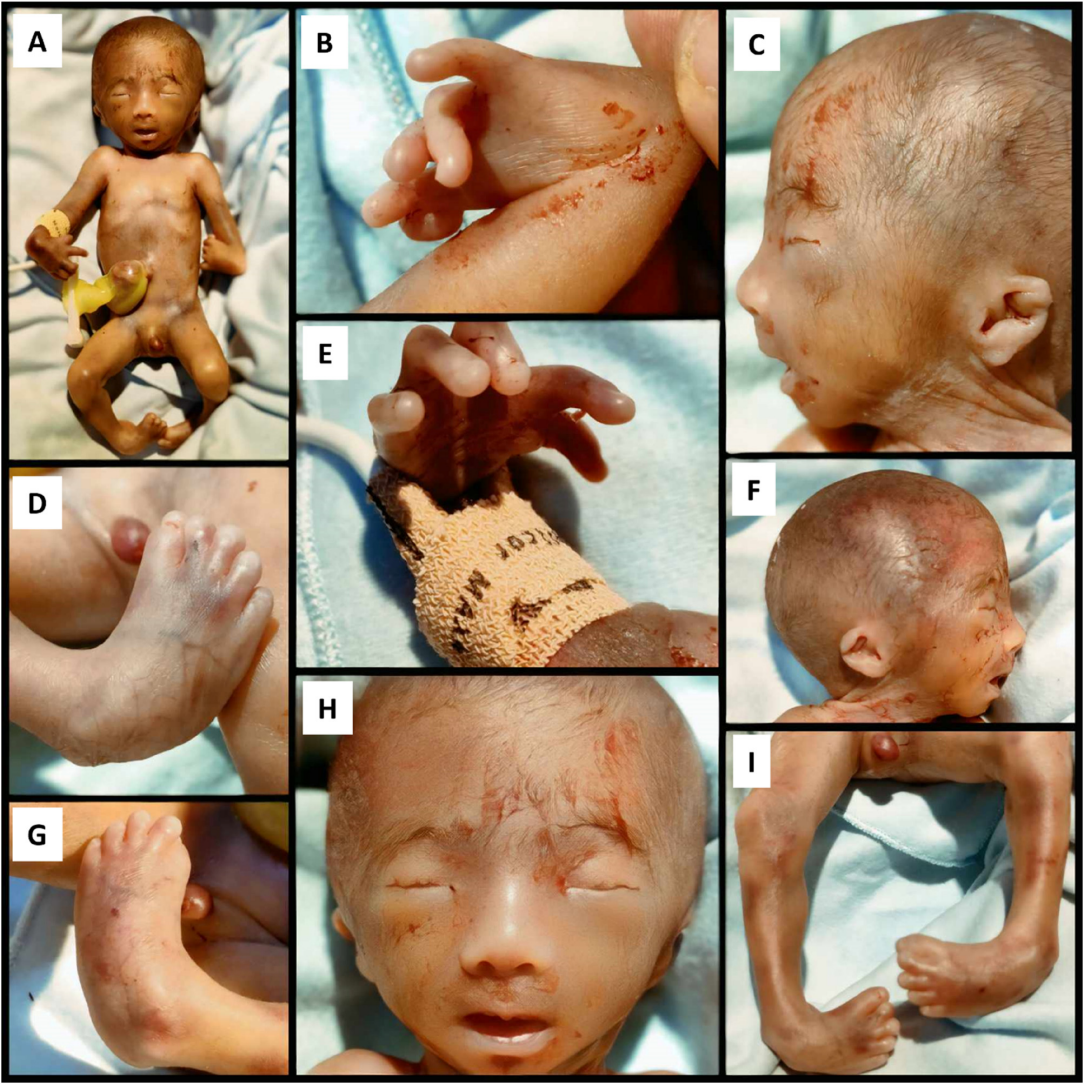
Provides a postnatal overview of a male neonate delivered at 26 weeks with confirmed syndromic Dandy-Walker malformation. The general view (A) reveals multiple congenital anomalies. Notable limb deformities include bilateral clubhands (B, E) and clubfeet (D, G, I), reflecting severe musculoskeletal involvement. Craniofacial features include low-set, dysplastic ears (C, F), and midface dysmorphism with a round face, flat nasal bridge, and hypotelorism (H). Panel (I) confirms male genitalia alongside bilateral lower limb deformities. These findings correlate strongly with prenatal imaging and underscore the multisystem nature of syndromic DWM.

At 26 weeks and 1 day, the patient presented with preterm premature rupture of membranes and entered spontaneous labor. A live male infant weighing 750 g was delivered vaginally. The neonate exhibited severe hypotonia, bradycardia, minimal spontaneous movement, and respiratory distress. Physical examination confirmed all prenatal anomalies: macrocephaly, bilateral clubhands and clubfeet, low-set dysplastic ears, and craniofacial dysmorphism. Postnatal images (Figure 4) corroborated prenatal findings. Despite resuscitation efforts, the infant died 2 h after birth. Per parental request, no autopsy was performed.

This case illustrates a rare and severe form of syndromic DWM with multisystem involvement. The application of KANET provided valuable insight into *in utero* neurobehavioral function and enabled accurate correlation with postnatal outcomes.

## Discussion

Dandy-Walker Malformation (DWM) is a complex congenital brain malformation characterized by hypoplasia or agenesis of the cerebellar vermis, cystic dilation of the fourth ventricle, and enlargement of the posterior fossa. These features are often accompanied by supratentorial anomalies, such as hydrocephalus and corpus callosum dysgenesis [2]. When DWM presents alongside extracranial anomalies – including cardiac, limb, and craniofacial defects, as in this case – it frequently suggests a syndromic or chromosomal etiology, even in the presence of a normal non-invasive prenatal test (NIPT). According to Stoll et al., over 70 % of fetuses with DWM exhibit additional anomalies, highlighting the need for comprehensive prenatal evaluation [1].

From a genetic standpoint, syndromic DWM has been associated with chromosomal deletions (13q, 3q, 6p) and syndromes such as Walker-Warburg and Meckel-Gruber. These typically involve midline brain defects, facial dysmorphism, and limb abnormalities. Although NIPT and standard karyotyping can rule out common aneuploidies, they may miss pathogenic microdeletions or single-gene mutations. Advanced methods such as chromosomal microarray analysis (CMA) or whole exome/genome sequencing (WES/WGS) could offer additional etiological insight. In our case, the unavailability of these tools and the family’s decision not to pursue invasive testing represent notable limitations.

The differential diagnosis of posterior fossa anomalies is essential, as prognosis varies considerably. Isolated DWM may carry a more favorable outlook, while syndromic forms – as seen here – are typically associated with worse outcomes. Although there was partial phenotypic overlap with VACTERL and CHARGE associations, the constellation of cerebellar hypoplasia, hydrocephalus, and microtia was most consistent with syndromic DWM.

The Kurjak Antenatal Neurodevelopmental Test (KANET) offers a unique approach for assessing fetal neurobehavior *in utero* using four-dimensional ultrasound. It evaluates a set of behavioral parameters – such as limb movements, facial expressions, and general posture – to provide a functional window into central nervous system (CNS) development [3], [4], [5], [6]. Though KANET is not a diagnostic test, numerous studies have shown its ability to predict neurodevelopmental impairment in high-risk pregnancies [8], [9], [10].

In our case, the fetus scored three on the KANET scale – well below the normal range of 10–16 – suggesting severe neurological dysfunction. Notable findings included fixed postures, clenched fists, lack of facial movement, and monotonous limb activity. These observations align with Prechtl’s concept that reduced movement complexity is a strong marker of CNS pathology [7]. AI-based image enhancement was utilized during the 4D ultrasound acquisition to improve contrast and minimize noise, facilitating the identification of subtle neurobehavioral features. This technological integration may offer added value in high-risk cases where fetal movements are minimal and difficult to interpret.

The postnatal outcome closely mirrored the antenatal findings: the neonate presented with severe hypotonia and respiratory insufficiency, confirming the prenatal prediction of poor neurological viability. This correlation reinforces the prognostic potential of KANET as a functional adjunct to anatomical imaging – especially in environments where fetal MRI or genetic testing may not be available.

Our findings echo those of Emir et al., who used KANET in a case of acephaly and noted preserved spinal activity. In contrast, the global behavioral suppression observed in our case points to extensive CNS dysfunction, likely exacerbated by associated cardiac anomalies such as hypoplastic left heart syndrome (HLHS) [6].

Although the Osaka Consensus recommends KANET application between 28 and 38 weeks of gestation [11], this window represents ideal conditions rather than strict limitations. Kurjak et al. [3], Honemeyer et al. [5], and Hata et al. [9] have all reported meaningful findings using KANET in the second trimester, particularly in structurally abnormal or high-risk fetuses. In our case, the early assessment at 25+3 weeks was clinically necessary, given the severity of anomalies and anticipated preterm delivery.

Despite limitations – including the absence of genetic confirmation and lack of autopsy – the integration of functional neurobehavioral testing added significant value to this prenatal evaluation. KANET served as a reliable screening tool, strengthening prognostic counseling and informing clinical decision-making in a context of syndromic DWM.

## Conclusions

This case highlights the integration of four-dimensional (4D) ultrasound and the Kurjak Antenatal Neurodevelopmental Test (KANET) as a valuable approach for assessing fetal neurobehavior in syndromic Dandy-Walker Malformation (DWM). While prenatal imaging identified severe structural anomalies, the markedly abnormal KANET score (3/16) provided critical insight into the fetus’s functional neurological status. Neurobehavioral deficits – such as absent facial expressions, reduced limb movements, and fixed postures – closely correlated with the adverse postnatal outcome.

The neonate’s rapid deterioration following delivery underscores the importance of incorporating functional assessments like KANET into the prenatal evaluation of complex CNS malformations. Despite limitations, including the lack of advanced genetic testing and postmortem examination, the findings illustrate KANET’s potential to augment structural imaging and improve prenatal counseling, particularly in resource-limited settings where fetal MRI is unavailable.

Ultimately, this case reinforces the concept that fetal behavior reflects central nervous system integrity. When used as part of a multidisciplinary evaluation, KANET offers a non-invasive means of detecting early signs of neurological impairment – helping guide prognosis, delivery planning, and perinatal decision-making in cases involving syndromic, multisystem fetal conditions.
